# The role of PKM2 nuclear translocation in the constant activation of the NF-κB signaling pathway in cancer-associated fibroblasts

**DOI:** 10.1038/s41419-021-03579-x

**Published:** 2021-03-17

**Authors:** Junjie Gu, Xuechun Li, Lin Zhao, Ying Yang, Chunling Xue, Yang Gao, Jing Li, Qin Han, Zhao Sun, Chunmei Bai, Robert Chunhua Zhao

**Affiliations:** 1 Department of Oncology, Peking Union Medical College Hospital, Chinese Academy of Medical Sciences and Peking Union Medical College, 100005 Beijing, China; 2 Institute of Basic Medical Sciences Chinese Academy of Medical Sciences, School of Basic Medicine Peking Union Medical College, Peking Union Medical College Hospital, Center of Excellence in Tissue Engineering Chinese Academy of Medical Sciences, Beijing Key Laboratory (No. BZO381), 100005 Beijing, China; 3 School of Life Sciences, Shanghai University, 99 Shangda Road, 200444 Shanghai, China

**Keywords:** Cancer microenvironment, Gastrointestinal cancer

## Abstract

Cancer-associated fibroblasts (CAFs) play critical roles in cancer progression by regulating tumor cell proliferation, angiogenesis, and metastasis. Recent studies demonstrated that CAFs induce inhibitory immune cell infiltration and chemotherapy resistance in gastric cancer by activating the NF-κB signaling pathway to secrete IL6, IL8, and other inflammatory factors. Inhibition of the NF-κB signaling pathway in CAFs might be a potential therapeutic strategy in gastric cancer. However, how the NF-κB pathway is activated in CAFs remains unclear. We showed that mesenchymal stem cells (MSCs) differentiated into CAFs, induced by the exosomes derived from gastric cancer cells. During the process of differentiation from MSCs into CAFs, we showed that nuclear PKM2 expression was continuously upregulated and associated with NF-κB P65 acetylation, contributing to P65 nuclear retention in CAFs and constant transcription of IL-6, IL-8, and other inflammatory factors, thus promoting gastric cancer cell proliferation. We showed that NF-κB P65 acetylation was induced by P300. We showed that nuclear PKM2 was derived from exosomes of gastric cancer cell lines and the positive feedback loop induced by PKM2-P65 combination. It is also proved that P300 inhibitors can inhibit tumor proliferation in an AGS subcutaneous xenograft tumor model. Our study showed that gastric cancer cells influence the continuous activation of the NF-κB signaling pathway in CAFs by secreting gastric cancer exosomes containing PKM2, thus inducing abnormal metabolism and inflammation activation. This study provides a new therapeutic target for CAF normalization or deactivation strategies.

## Introduction

Gastric cancer is the third leading cause of cancer-related death worldwide. The overall survival time of patients with advanced gastric cancer is usually <1 year, and the standard treatment remains to be the adjuvant chemotherapy^1,2^. Over the past 50 years, the research on cancer treatment has predominantly focused on targeting tumor cells. However, with the growth in knowledge of tumor microenvironment (TME), more studies have focused on revealing the incredible complexity of the TME and determining therapeutic TME targets. Many nontumor cells are recruited into the tumor tissue. Then nontumor cells are transformed, activated, and reprogrammed into pro-tumorigenic cells by cancer cells. These pro-tumorigenic cells can secrete numerous inflammatory factors, growth factors, chemokines, and extracellular matrix proteins and thus create a protective microenvironment to promote tumor progression and metastasis^3^. Cancer-associated fibroblasts (CAFs) are one of the most abundant nontumor components in the TME. CAFs play a prominent role in enhancing gastric cancer cell proliferation, angiogenesis, migration, and invasion and in promoting inhibitory immune cell infiltration, thereby leading to chemotherapy resistance in gastric cancer^3^.

Various anticancer therapies targeting CAFs have been reported. There are two main practices: direct CAF depletion via surface markers and normalization of CAFs^4^. Direct CAF depletion relies on targeting unique CAF markers. However, CAFs are highly heterogeneous, depending on their origins. Therefore, it is difficult to practice direct CAF depletion. Normalization of CAFs, via suppressing the tumor-promoting effective molecules and CAF-activated signaling pathways, seems to be more practical. It is thus important to investigate the mechanism of abnormal activation of CAFs to screen an appropriate therapeutic target.

The nuclear factor (NF)-κB signaling pathway plays a vital role in promoting the expression of inflammatory cytokines and chemokines. The constant activation of the NF-κB signaling pathway in CAFs, identified in different tumors, promotes tumor progression via producing interleukin (IL)-6 and IL-8. Su et al. first reported that the continuous activation of the NF-κB signaling pathway in CAFs can promote IL-6 and IL-8 expression in breast cancer and lung cancer patients, which plays a pivotal role in maintaining the stemness of cancer stem cells, leading to chemotherapy resistance^5^. Zhai et al. reported that IL-8 secreted by CAFs induced cisplatin resistance in gastric cancer cells. Ham et al. reported that IL-6 secreted by CAFs suppressed gastric cancer cell apoptosis induced by chemotherapy. IL-6 secreted by CAFs could promote tumor epithelial–mesenchymal transition in bladder cancer cells^6^. C-C chemokine motif ligand 2, IL-6, and C-X-C chemokine motif ligand 8 secreted by CAFs can actively promote the recruitment of macrophages to the TME and their differentiation toward M2 macrophages in esophageal squamous cell carcinoma^7^. However, the underlying mechanism of how can CAFs maintain the continuous activation of the NF-κB signaling pathway remains unraveled.

Deregulating cellular energetics is one of the hallmarks of cancer^8^. Recent studies have shown that CAFs also favor aerobic glycolysis in the TME under normoxic conditions^9^, which is known as the reverse Warburg effect^10^. Previous studies on the reverse Warburg effect mainly focused on the provision of energy-rich metabolites to cancer cells; however, metabolic reprogramming may also lead to other functional changes in CAFs. In the in vitro model of CAFs differentiated from mesenchymal stem cells (MSCs) by gastric cancer-derived exosomes, we found that the main protein components of gastric cancer-derived exosomes are metabolism-related proteins, especially glycolytic enzymes, M2 pyruvate kinase (PKM2), lactate dehydrogenase A (LDHA), and phosphofructokinase P (PFKP), while PKM2 was frequently upregulated at the mRNA and protein levels in CAFs differentiated from MSCs. PKM2 in the cytoplasm is a rate-limiting enzyme for glycolysis. In the nucleus, PKM2 functions as a protein kinase and a transcriptional co-activator of many genes associated with cancer cell proliferation, metastasis, and apoptosis^11^. For example, it can promote cancer cell metastasis by upregulating matrix metalloproteinase-2 (MMP2), MMP9, N-cadherin, and vimentin and can promote tumor cell growth by upregulating β-catenin expression^12,13^. PKM2 is also involved in the regulation of the NF-κB signaling pathway in cancer cells. Azoitei et al. found that PKM2 triggered vascular endothelial growth factor-A secretion through the activation of NF-κB and hypoxia-inducible factor (HIF)-1α and subsequently influenced tumor angiogenesis in pancreatic cancer^14^. Zheng et al. reported that PKM2 could promote the translocation of NF-κB/P65 to the nucleus and thus induce cancer cell migration and invasion in ovarian cancer^15^. PKM2 is also overexpressed in other cells in the TME and affects the activation of T and B cells, the immune response of macrophages and dendritic cells, and the recruitment of myeloid-derived suppressor cells in tumors^12^.

CAFs are a highly heterogeneous population, which is derived from different cell types^16^. Tissue-derived fibroblasts or MSCs are considered as the main source of CAFs, and it is believed that MSCs are inactivated fibroblasts^17^. Because MSCs are relatively easy to obtain, CAFs differentiated from MSCs by cancer exosomes or co-culture with cancer cells are good in vitro models for studying the functions and signaling pathways of CAFs. We previously found that gastric cancer exosomes could promote the differentiation of MSCs into CAFs and induce the continuous activation of the NF-κB signaling pathway in CAFs, subsequently leading to the secretion of inflammatory factors. However, the protein content and phosphorylation level of the NF-κB canonical signaling pathway hardly changed. A large amount of PKM2 was found in gastric cancer exosomes. PKM2 could be transferred from gastric cancer cells into CAFs through gastric cancer exosomes. In addition to determining the effect on the metabolism of CAFs, we investigated whether PKM2 is involved in the constant activation of the NF-κB signaling pathway in CAFs.

## Materials and methods

### Mice

All nude mice (female, 8 weeks) were purchased from the Laboratory Animal Center of the Chinese Academy of Medical Sciences (Beijing, China). All mice were maintained in a specific pathogen-free facility, and all animal experiments were conducted under the protocols approved by the Animal Care and Use Committee of the Chinese Academy of Medical Sciences.

### Cell cultures

Adipose tissues were obtained from patients undergoing liposuction according to procedures approved by the Ethics Committee of the Chinese Academy of Medical Sciences and Peking Union Medical College. All the donors provided written informed consent. MSCs were isolated, cultured, and propagated as previously reported^18,19^. MSCs at passage 3–4 were used for all the experiments.

AGS and GC803 gastric cancer cell lines were purchased from the Cell Resource Center at the Chinese Academy of Medical Sciences and were authenticated by the Cell Resource Center. We tested that there is no mycoplasma contamination during cell culture. The AGS cells were cultured in Dulbecco’s modified Eagle’s medium (DMEM)/F12 (Gibco, Paisley, UK) with 10% fetal calf serum (FCS) (fetal bovine serum (FBS), Gibco, Paisley, UK), penicillin (100 U/ml), and streptomycin (100 μg/ml) at 37 °C in a humidified incubator with 5% CO_2_. The GC803 cells were cultured in DMEM (Gibco, Paisley, UK) with 10% FCS (FBS, Gibco, Paisley, UK), penicillin (100 U/ml), and streptomycin (100 μg/ml) at 37 °C in a humidified incubator with 5% CO_2_.

### Identification of MSC surface markers by FACS

The surface markers of third-generation adipose-derived MSCs (hAD-MSCs) were identified by fluorescence-activated cell sorting (FACS). The cells were washed, centrifuged, and resuspended in samples as previously reported^18^. Then 50 µl of 1:100 dilution of mouse anti-human primary antibodies CD29, CD44, CD31, CD34, CD90, CD105, and HLA-DR were added to the samples. The samples were incubated at 4 °C for 30 min and washed twice with phosphate-buffered saline (PBS). The cells were incubated with fluorescein isothiocyanate and phycoerythrin-conjugated secondary antibodies for 30 min at 4 °C and washed twice with PBS. The control sample was incubated only with secondary antibodies. The samples were analyzed using the BD Accuri C6.

### Osteogenic and adipogenic differentiation

The osteogenic and adipogenic differentiation was conducted as previously described^20^. The culture-expanded hAD-MSCs at 2 × 10^4^/cm^2^ were induced in the osteogenic medium for 2 weeks: DMEM with 10% FBS, 10^−^^7^ mol/l dexamethasone, 0.2 mmol/l ascorbic acid, and 10 mmol/l β-glycerophosphate (all from Sigma-Aldrich). The cells were then stained with alkaline phosphatase to reveal osteogenic differentiation. The culture-propagated cells at 2 × 10^4^/cm^2^ were induced for 2 weeks in DMEM with 10% FCS, 0.5 μmol/l hydrocortisone, 50 μg/ml indomethacin, and 0.5 mmol/l isobutyl methylxanthine (all from Sigma-Aldrich). The cells were fixed in 10% formalin for 10 min and then stained with fresh Oil Red O solution (Sigma-Aldrich) to present lipid droplets in the induced cells.

### Extraction and identification of exosomes

Exosome extraction was conducted as previously described^18^. The AGS medium was replaced with FBS-free DMEM/F12 medium, and the GC803 medium was replaced with the FCS-free DMEM medium 24 h before exosome extraction. After culturing for 24 h, culture supernatant was collected and centrifuged at 1500 rpm for 20 min to discard nonviable and dead cells. A 0.22-μm microporous filter was used to filter the supernatant, and the remaining large vesicles and cell debris were discarded by filtration. The filtered supernatant was then transferred to a 100,000 molecular weight ultrafiltration membrane, and an ultrafiltration process was conducted at 4 °C until the supernatant was completely centrifuged. An appropriate amount of PBS solution was used to wash the sample twice, and the resultant liquid was transparent and colorless. Approximately 1 ml of the resultant liquid was pipetted repeatedly, transferred to a 1.5-ml EP tube, and filtered with a 0.2-µm Millipore filter. Then, 200 μl of the sample was dispensed into a 1.5-ml sterile EP tube and stored at −80 °C. In the experiments involving exosomes, we used PBS as a blank control.

Exosomes were identified by transmission electron microscopy as previously described^18^. The purified exosomes were first diluted and precipitated on a copper grid for 5 min. Excess liquid was absorbed by a filter paper. The exosomes were dried and diluted with 3% aqueous phosphotungstic acid for 2 min. Transmission electron microscopy was used to analyze and photograph the exosomes. The uniformity and size of the exosomes extracted from the AGS (AGS-Exos) and GC803 (GC803-Exos) gastric cancer cell lines were identified using Nanosight (Zetasizer Nano ZS90). After diluting the AGS-Exos and GC803-Exos, an appropriate amount of each sample was added to transparent tubes to measure the particle size on the machine. The western blotting assay was performed to show the protein expression of the gastric cancer cell exosomes.

### Plasmids and virus infection

PKM2-eGFP fusion genes were designed and synthesized by GenePharma (Suzhou, China) for PKM2 overexpression. The full-length PKM2-eGFP fusion or eGFP gene was inserted into the lentivirus expression vector pEZ-LV22-puro, termed LV-PKM2-eGFP or LV-eGFP. The lentiviral particles were generated according to a standardized protocol by using highly purified plasmids, EndoFectin-Lenti, and TiterBoost reagents obtained from GenePharma. The lentivirus particles were purified and stored at −80 °C in aliquots (purified particles).

### Exosome labeling and uptake experiments

MSCs were transfected with the eGFP gene using the lentivirus vector LV-eGFP and were termed as eGFP-MSCs. Then, 1 mM Dil was added to the purified AGS exosomes, stained for 10 min, and washed twice by ultrahigh-speed centrifugation (700,000 × *g* at 4 °C for 40 min). The supernatant was discarded. Dil exosomes were resuspended in DMEM/F12 and co-incubated with eGFP-MSCs. After 24 h, the medium was discarded, washed three times with PBS, fixed in 4% paraformaldehyde for 10 min, stained with Hoechst 33342 (1:1,000 dilution), washed at room temperature for 5 min, washed three times with PBS, and observed under a confocal microscope (Olympus).

### Subcellular fractionation

The nuclear and cytoplasmic fractions were separated using NE-PER nuclear and cytoplasmic extraction reagents (#78833, Thermo Fisher Scientific) according to the manufacturer’s instructions. Protein was extracted, and the western blotting assay was performed to assess the relative protein content in the nuclear and cytoplasmic fractions.

### Western blotting

Western blotting assay was conducted as previously described^21^. After washing twice with cold PBS, the cells were lysed in RIPA lysis buffer (Beyotime) with 1 mM PMSF and a protease inhibitor cocktail on ice for 30 min; the cells were then manually scraped from the culture plates and quantified using a BCA protein assay kit (Beyotime). Proteins were separated on 10% sodium dodecyl sulfate-polyacrylamide gel electrophoresis gels and electroblotted onto a polyvinylidene difluoride membrane (0.2 μm, Millipore). The membranes were blocked with 3% bovine serum albumin (BSA), incubated with specific antibodies overnight at 4 °C, and further incubated with horseradish peroxidase-conjugated secondary antibody (anti-rabbit IgG, #7074, and anti-mouse IgG, #7076; CST, Danvers, MA, USA) for 1 h at room temperature. The primary antibodies were as follows: GAPDH (#5174, CST), PKM2 (#4053, CST), pyruvate dehydrogenase (#3205, CST), hexokinase I (#2020, CST), hexokinase II (#2867, CST), LDHA (#3582, CST), PKM1/2 (#3190, CST), PFKP (#8164, CST), IKKβ (#8943, CST), phospho-IKKα/β (Ser176/180, #2697, CST), phospho-NF-κB P65 (Ser536, #3033, CST), IκBα (#4814, CST), phospho-IκBα (Ser32, #2859, CST), NF-κB P65 (#8242, CST), P300 (#86377, CST), acetyl-NF-κB P65 (Lys310, #12629, CST), α-smooth muscle actin (#19245, CST), FAP (#66562, CST), IL-6 (#12912, CST), lamin B1 (#13435, CST), IL-1β (#12703, CST), IL-8 (#17038-1-AP, Proteintech, Rosemont, IL, USA), CD63 (#10638D, Thermo Fisher, Waltham, MA, USA), Hsp70 (#4873, CST), Hsp90 (#4877, CST), and β-actin (#4970, CST).

Antibody and antigen complexes were detected using a chemiluminescent ECL reagent (Millipore).

### RNA extraction and quantitative reverse transcription–polymerase chain reaction (qRT-PCR)

Total RNA was extracted using TRIzol reagent (15596018, Thermo Fisher), and qRT-PCR analysis of mRNA was conducted as previously described^19^. All the primer sequences are listed in Supplementary Table 1.

### Chromatin immunoprecipitation (ChIP) assay

ChIP assay was performed using a chromatin immunoprecipitation kit (SimpleChIP enzymatic chromatin IP kit, magnetic beads, #9003, CST) following the manufacturer’s instructions. The antibody used was NF-κB P65 antibody (#8242 CST) and IgG isotype control (#3900, CST). The primer of the PKM2 promoter is shown in Fig. 5A.

### Co-immunoprecipitation (Co-IP)

Co-IP was conducted using an immunoprecipitation kit (ab206996, Abcam, Cambridge, UK) following the manufacturer’s instructions. The following Co-IP antibodies were used: NF-κB P65 (#8242, CST), PKM2 (#4053, CST), P300 (#86377, CST), and IgG isotype control (#3900, CST).

### Immunofluorescence (IF) staining

IF staining was conducted as previously described^22^. The cultured cells were fixed at 4 °C in ice-cold methanol for 10 min, washed three times in PBS, and then permeabilized in 0.1% Triton X-100/PBS for 10 min at room temperature. Nonspecific binding was blocked with 0.5% Tween-20/PBS containing 3% BSA for 30 min. The cells were incubated with primary antibodies (#436700, NF-κB P65, Thermo Fisher; #4053, PKM2, CST) at 4 °C overnight. The cells were then incubated with secondary antibodies (#A-11078, Alexa Fluor 488 goat anti-rabbit IgG, #31660, rhodamine goat anti-mouse IgG, Thermo Fisher) for 1 h at room temperature. The incubated cells were washed with PBS, and Hoechst 33342 (#H1399, Thermo Fisher) was used to visualize the nuclei. The cells were incubated at room temperature for 5 min, washed three times with PBS, and observed under a confocal microscope (Olympus).

### Proteomic analysis of gastric cancer exosomes

AGS-Exo, GC803-Exo, and MSC-Exo were subjected to mass spectrometry (MS). Proteins were extracted in a urea-containing buffer by using a sonication bath. Urinary proteins were digested with trypsin (Trypsin Gold, mass. spec. grade, Promega, Madison, WI, USA) by using filter-aided sample preparation methods [30894400]. Each sample was analyzed on a reverse-phase C18 self-packed capillary LC column (75 μm × 100 mm, 3 μm). The elution gradient used was 5–30% buffer B2 (0.1% formic acid and 99.9% acetonitrile; flow rate = 0.3 μl/min) for 100 min. A TripleTOF 5600 mass spectrometer coupled with a UPLC system was used to analyze the sample. The MS data were acquired in the high sensitivity mode^23^. Data analysis was conducted by Kang Chen Biotechnology. The gastric cancer-specific exosomal protein content was determined by subtracting the exosomal protein content of AGS from that of MSCs.

### Cell proliferation assay

MSCs or CAFs were cultured by DMEM/F12 with 10% FBS for 24 h. The culture supernatant was collected as a conditioned medium. The supernatants were centrifuged at 1500 rpm for 20 min to remove nonviable and dead cells (called MSC-CM or CAF-CM). AGS were plated in 96-well plates (2000 cells/well) and cultured in DMEM/F12 with 10% FBS, MSC-CM, or CAF-CM, respectively. Cell proliferation was determined every 24 h for 3 days according to the manufacturer’s instructions. Briefly, 20 μl of MTS (#G3582, Promega) was added to each well. After incubation at 37 °C for 1 h, the absorbance was detected at 490 nm.

### NF-κB activation assay

The NF-κB activation assay was performed using TransAM NF-κB activation assay colorimetric kits (#40096, Active Motif, Carlsbad, CA, USA). The Kit detected the activated form of NF-κB by a consensus oligonucleotide sequence shared with NF-κB targeted genes. The positive control is the Raji nuclear extract provided in the kit. The negative control is the complete lysis buffer without cell extracts.

### Xenograft assay in nude mice

To develop the AGS subcutaneous xenograft tumor model, female Balb/c nude mice (5–6 weeks old) were subcutaneously injected with 100 μl of cell suspension containing either 5 × 10^6^ AGS cells or 5 × 10^6^ AGS cells mixed with 1 × 10^6^ MSCs. From the second week, the mice in the MSC mixed AGS group were subcutaneously injected with C646 (1.5 µg/75 µl) or dimethyl sulfoxide (75 µl) around the tumor every 2 days. The tumor sizes were measured every week for 5 weeks, and the tumor volume (in cubic millimeters) was calculated according to the equation: width^2^ × length × 0.5.

### Statistical analysis

Data are presented as mean ± SD. Comparisons between the groups were analyzed using *t* test by SPSS 19.0. Differences were considered to be statistically significant at *P* < 0.05. Sample sizes of all experiments were predetermined based on published literatures and our experience. No sample was excluded from the analyses. Animals were randomly assigned during collection. The strain, sex, and age of the mice were the same. The investigator was not blinded to the group allocation. The data analysis is single blinded. The number of replicates is indicated in the figure legend.

## Results

### Gastric cancer exosomes induced differentiation of MSCs into CAFs

**Fig. 1:**
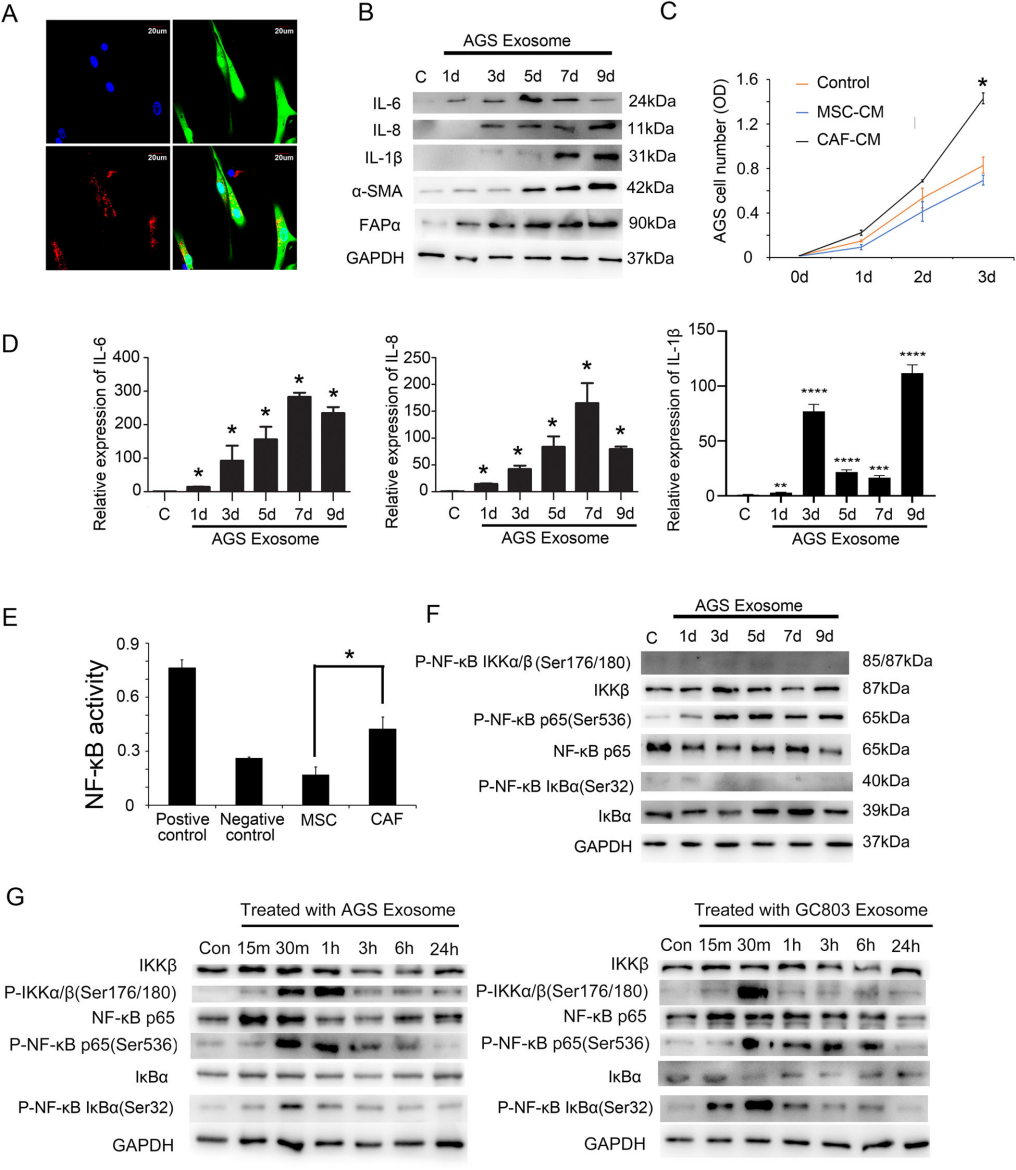
Gastric cancer exosomes induced differentiation of MSCs into CAFs. **A** Confocal microscopy detected that MSCs (eGFP-Green) could take up gastric cancer exosomes (Dil-Exosome) into the cytoplasm; **B** western blotting analysis showed that the expression of CAF-specific biomarkers and inflammatory factors was upregulated after treatment with AGS exosomes. **C** CAF-conditioned medium promoted AGS proliferation, which was significantly different from the AGS control group. Control group: AGS culture medium, MSC-CM: MSC conditioned medium, CAF-CM: CAF conditioned medium. *P < 0.05. **D** Quantitative PCR analysis detected that the expression of inflammatory factors and chemokine in CAFs differentiated from MSCs after treatment with AGS exosomes. *P < 0.05, **P < 0.01, ***P < 0.001, ****P < 0.0001. **E** NF-κB transcriptional activity increased when MSCs were induced to differentiate into CAFs by AGS exosomes. *P < 0.05. **F** Western blotting analysis detected the NF-κB signaling pathway protein expression in CAFs differentiated from MSCs after treatment with AGS exosomes on days 1, 3, 5, 7, and 9. **G** Western blotting analysis detected the NF-κB signaling pathway protein expression in CAFs differentiated from MSCs after treatment with AGS exosomes at 15 min, 30 min, 1 h, 3 h, 6 h, and 24 h.

To investigate whether gastric cancer cells can secrete exosomes to induce the differentiation from MSCs to CAFs, we purified exosomes of gastric cancer cell lines to induce MSCs. MSCs were harvested from the adipose tissue of healthy donors. We showed that the isolated cells expressed CD29, CD44, CD90, CD105, and other specific biomarkers of MSCs (Supplementary Fig. 1). Gastric cancer exosomes were extracted from the culture supernatant of gastric cancer cell lines GC803 and AGS. We showed that gastric cancer exosomes presented the double-layer membrane vesicle-like structures, with average sizes ranging from 40 to 100 nm, using transmission electron microscopy (Supplementary Fig. 2A). The expression of exosome markers, such as CD63, Hsp70, and Hsp90, were identified by western blot (Supplementary Fig. 2B). To investigate whether gastric cancer exosomes can be taken up by MSCs, AGS exosomes were first stained with Dil and then added to the eGFP-MSC culture medium. After incubation, we showed that Dil-stained AGS exosomes accumulated in MSCs. (Fig. 1A). After 9 days of culture, western blotting assay showed that the expression of α-SMA and FAPα in the gastric cancer exosome-added MSC group was gradually upregulated compared to that in the MSC control group (Fig. 1B). We also found that the multilineage differentiation potential of MSCs disappeared (Supplementary Fig. 3). These findings confirmed that MSCs were differentiated into CAFs by gastric cancer exosomes.

To examine whether these induced CAFs gain the pro-tumor abilities, we first compared the effects of CAFs and MSCs on the proliferation of gastric cancer cells (AGS). We treated AGS with the conditioned medium derived from CAFs (CAF-CM) or the conditioned medium derived from MSCs (MSC-CM). We showed that AGS treated with CAF-CM presented a higher proliferative rate than the untreated control group (Fig. 1C). AGS treated with MSC-CM presented a lower proliferative rate than the untreated control group (Fig. 1C). This result indicated that induced CAFs promote the proliferation of gastric cancer cells, differing from their precursors, MSC. We also investigated whether CAFs upregulated the production of inflammatory factors, which play vital roles in promoting tumor progression. On days 0, 1, 3, 5, 7, and 9 after CAFs were induced by gastric cancer exosomes, we detected the expression of inflammatory factors. We found that the expression levels of IL-1B, IL-6, and IL-8 upregulated at the protein level (Fig. 1B and Supplementary Fig. 4A) and mRNA level (Fig. 1D and Supplementary Fig. 4B) constantly after 9-day treatment compared to the untreated control group. However, the expression of IL-6 dropped on days 7 and 9. We estimated that it may be the results of posttranscriptional regulation or the secreting of IL-6 from the cytoplasm. The elevated expression of inflammatory factors in CAFs was significantly different from that noted for MSCs. The transcription of these inflammatory factors depends on the activation of the NF-κB signaling pathway. Therefore, we examined NF-κB transcriptional activity. The NF-κB transcriptional activity of CAFs also increased significantly compared to that of MSCs (Fig. 1E). To investigate the mechanism underlying the constant activation of the NF-κB signaling pathway, we detected the expression levels of the key proteins involved in the activation of the NF-κB signaling pathway during the 9-day treatment process. We showed that the expression of NF-κB P65 (P-ser536) increased after the addition of gastric cancer exosomes to the MSC culture system (Fig. 1F). We showed that the phosphorylated levels of IKKα/β and IκBα increased after treating with gastric cancer cell exosomes at 30 min but decreased at 24 h (Fig. 1G). Furthermore, the total expression levels and the phosphorylated levels of these two proteins did not increase on days 1, 3, 5, 7, and 9 (Fig. 1F and Supplementary Fig. 4C). These results indicated that the constant activation of the NF-κB signaling pathway in CAFs was not induced by the canonical pathway. Therefore, the underlying mechanism of the constant activation of the NF-κB signaling pathway in CAFs needs to be further investigated.

### The main components of gastric cancer exosomes

**Fig. 2:**
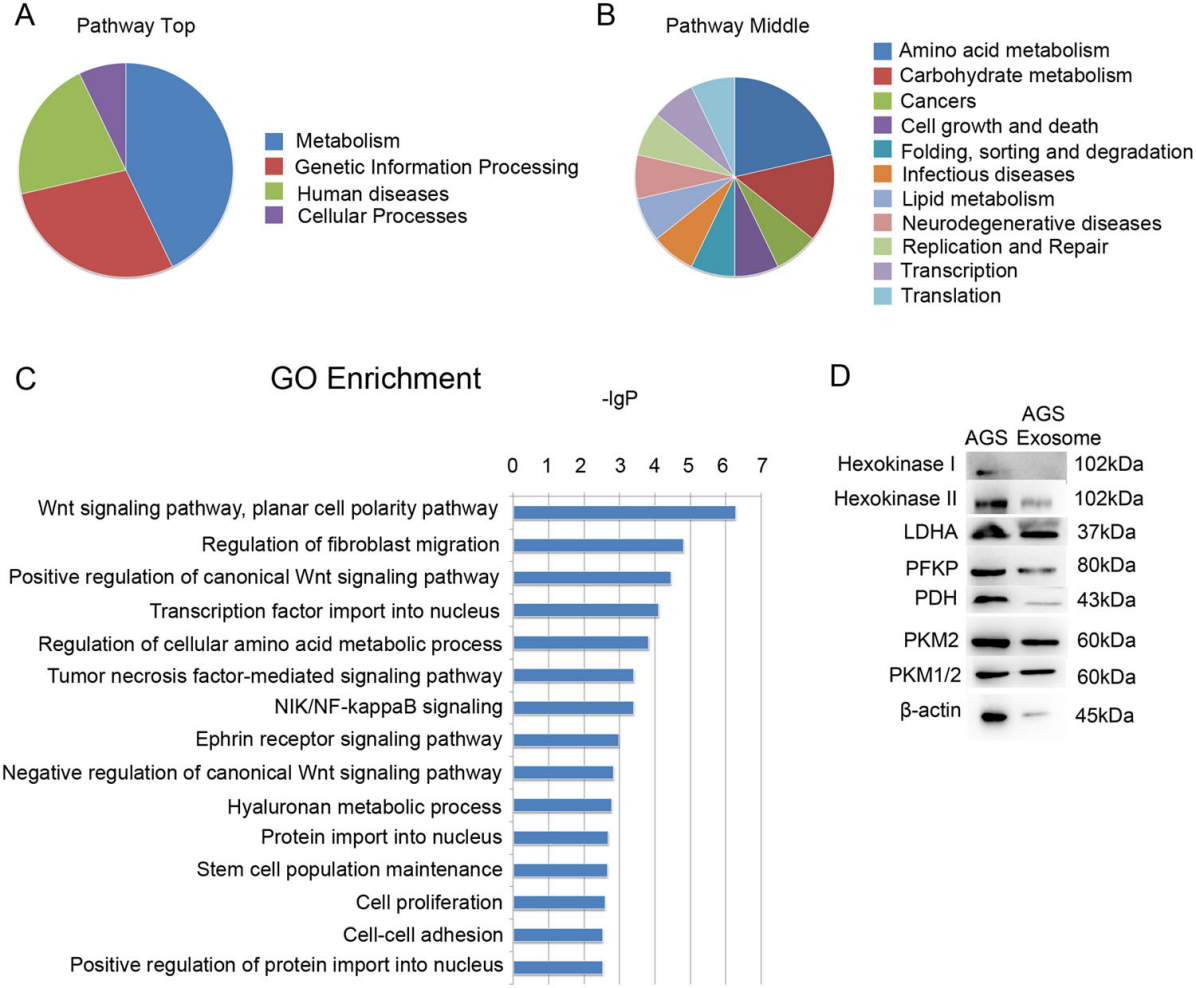
The protein components of gastric cancer exosomes. **A**, **B** Pathway enrichment analysis of gastric cancer exosomes. **A** Pathway top. **B** Pathway middle. **C** GO enrichment analysis of gastric cancer exosomes. **D** Glycolysis enzymes of AGS and AGS exosomes by western blotting.

To further analyze how gastric cancer exosomes affect the differentiation of MSCs, a protein profile was established to determine the protein components of exosomes derived from gastric cancer cell lines (GC-803 and AGS) and MSC. Gene Ontology and Kyoto Encyclopedia of Genes and Genomes analyses were conducted to analyze the specific protein components of gastric cancer exosomes (proteins contained in gastric cancer exosomes minus proteins contained in MSC exosomes). The results showed that the signaling pathways mainly involved in these proteins were related to amino acid metabolism, glycolysis, pyruvate metabolism, and lipid metabolism (Fig. 2A, B). The new signaling pathways that involved these proteins included Wnt, fibroblast migration, tumor necrosis factor (TNF)-α and NF-κB signaling pathways (Fig. 2C). This finding suggested that gastric cancer exosomes were mainly associated with protein metabolism and could promote the differentiation of MSCs into CAFs by promoting the energy metabolism of MSCs. We then verified the protein profile results by western blotting assay and found that gastric cancer exosomes contained key enzymes of the glycolysis pathway, such as PKM2, LDHA, and PFKP (Fig. 2D).

### PKM2 and P65 were simultaneously upregulated in the nucleus during the differentiation of MSCs into CAFs

**Fig. 3:**
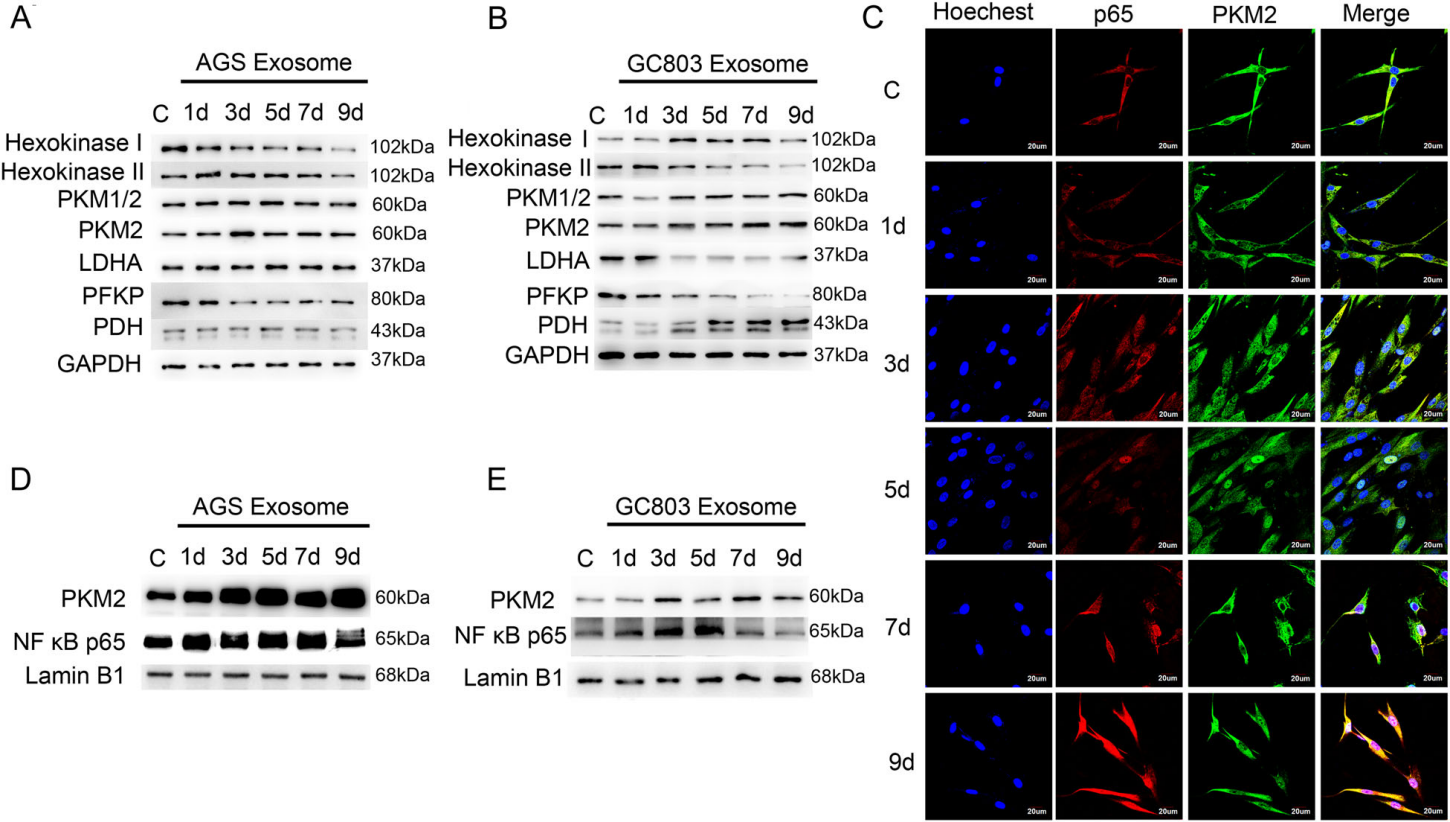
PKM2 and P65 in the nucleus were simultaneously upregulated during the differentiation of MSCs into CAFs induced by gastric cancer exosomes. **A**, **B** The protein-level changes of glycolysis enzymes (Hexokinase I, Hexokinase II, PKM1/2, PKM2, LDHA, PFKP, and PDH) during the differentiation of MSCs into CAFs induced by AGS exosomes (**A**) or GC803 exosomes (**B**) were evaluated by western blot. **C** Confocal microscopy detected PKM2 and P65 expression in the nucleus during the differentiation of MSCs into CAFs induced by AGS exosomes. Blue: Nucleus (Hoechst 33342). Red: P65 (rhodamine). Green: PKM2 (Alexa Fluor 488. **D**, **E** Western blotting analysis detected PKM2 and P65 changes in the nucleus.

Tumor cells are reported to promote the dependency of CAFs on glycolysis. We therefore estimated that key enzymes of the glycolysis pathway could upregulate during the differentiation of MSCs into CAFs. Therefore, we detected changes in the key enzyme of glycolysis during the differentiation of MSCs into CAFs and found that PKM2 was upregulated in CAFs (Fig. 3A, B). According to previous reports^13^, PKM2 plays an important role in protein modification and transcriptional regulation after nucleus translocation, and the constant transcription of the NF-κB signaling pathway depends on P65 nuclear retention. Therefore, we planned to verify the relationship between PKM2 nuclear translocation and P65 nuclear retention. First, we examined the correlation between PKM2 and P65 expression levels during the differentiation of MSCs into CAFs. The control group MSCs obtained on days 1, 3, 5, 7, and 9 after CAFs were induced by gastric cancer (GC803 and AGS) exosomes were fixed and labeled with rhodamine (red) for P65, Alexa Fluor 488 (green) for PKM2, and Hoechst 33342 (blue) for the nucleus. Confocal microscopy showed that P65 and PKM2 were localized in the cytoplasm of MSCs, and they gradually entered the nucleus after the addition of gastric cancer exosomes (Fig. 3C and Supplementary Fig. 5). We then performed cytoplasmic and nuclear extraction in MSCs on days 1, 3, 5, 7, and 9 after CAFs were induced by gastric cancer exosomes. The western blotting analysis revealed the expression of PKM2 and P65 in the nucleus and showed that the expression levels of PKM2 and P65 gradually increased with the progression in the induction time (Fig. 3D, E).

**Fig. 4:**
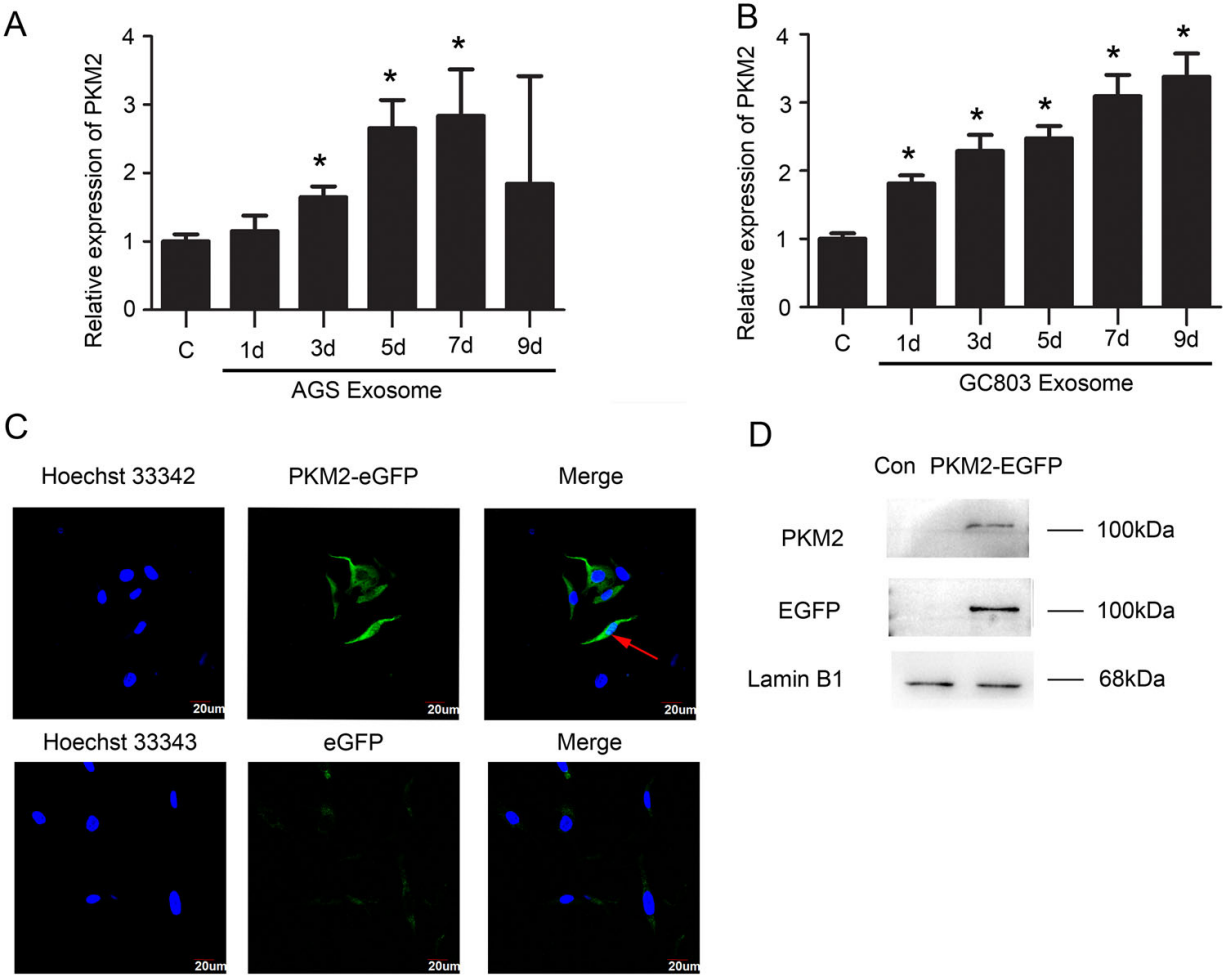
Sources of upregulated PKM2 in CAFs. **A**, **B** Quantitative PCR analysis detected PKM2 expression during the differentiation of MSCs into CAFs. *P < 0.05. **C** Confocal microscopy detected PKM2-eGFP protein of AGS could be recruited to the cytoplasm and nucleus of MSCs/CAFs. Blue: Nucleus (Hoechst 33342). Green: PKM2-eGFP fusion protein or EGFP-vector. **D** Western blot of the expression of the PKM2-eGFP fusion protein in the nucleus.

Correlating to the result that the exosomes derived from gastric cancer cells include PKM2, we estimated that the upregulated PKM2 in CAFs had two sources: external PKM2 present in gastric cancer exosomes and endogenous PKM2 expression induced by gastric cancer exosomes. To verify our estimations, we detected the transcription levels of the *PKM2* gene during the differentiation from MSCs to CAFs on days 1, 3, 5, 7 and 9 by qPCR. We found that PKM2 mRNA was upregulated as the induction time progressed, with 3–4-fold higher expression than the untreated control group (Fig. 4A, B), indicating that endogenous PKM2 transcription can be induced by gastric cancer cells via exosomes. To investigate whether external PKM2 in the exosomes contributes to the accumulation of PKM2 in CAFs, we traced the moving track of PKM2 from exosomes into CAFs. We detected the nucleus translocation of external PKM2 after treating MSC with PKM2-EGFP-transfected AGS exosomes using IF and western blot. We showed that external PKM2 were recruited into the cytoplasm and translocated into the nucleus of CAFs via exosomes (Fig. 4C, D). Thus, the upregulated PKM2 during the differentiation of MSCs into CAFs was derived from both PKM2 present in gastric cancer exosomes and endogenous PKM2 expression.

### Mechanism of promotion of NF-κB P65 retention by PKM2 in the nucleus

**Fig. 5:**
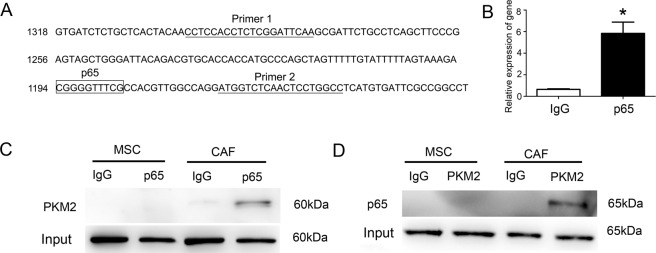
The interaction between PKM2 and P65. **A** The PKM2 promoter region had a binding box of P65. **B** Quantitative PCR analysis detected the chromatin–protein complex captured by CHIP P65 antibody containing the PKM2 promoter region sequence. *P < 0.05. **C** Western blotting analysis detected the whole-cell lysate protein captured by Co-IP P65 antibody containing PKM2 protein. **D** Western blotting analysis detected cell lysate protein captured by Co-IP PKM2 antibody containing P65 protein.

**Fig. 6:**
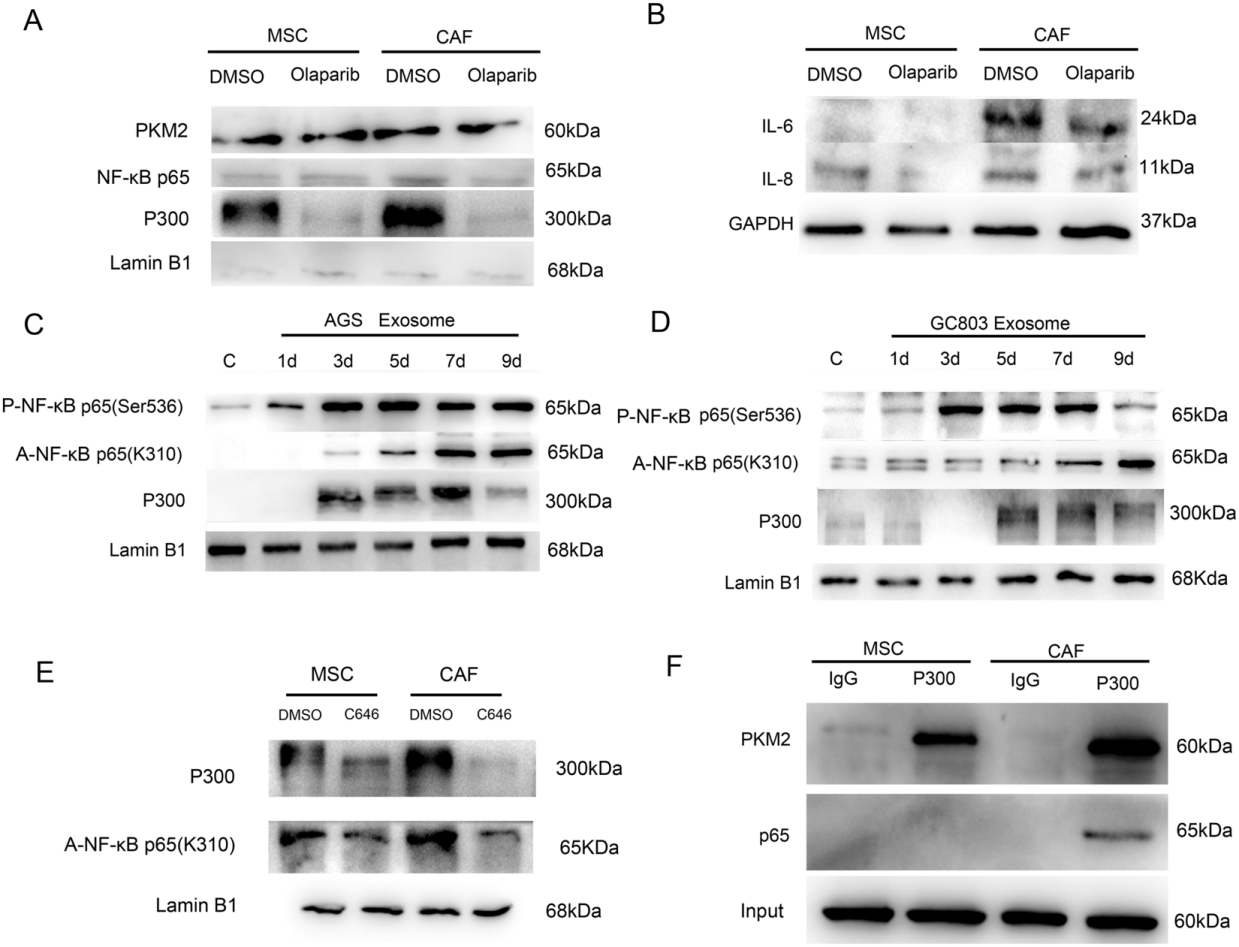
Mechanism of PKM2 promoting NF-κB P65 retention in the nucleus. **A** Western blotting detected nuclear protein expression in CAFs(AGS exosomes-treated) after adding PKM2 nuclear translocation inhibitor olaparib. **B** Western blotting detected inflammatory factor expression in CAFs (AGS exosome treated) after the addition of the PKM2 nuclear translocation inhibitor olaparib. **C**, **D** Western blotting detected nuclear protein expression during the differentiation of MSCs into CAFs. **E** Acetylated P65 expression in the nucleus of MSCs and CAFs (AGS exosome treated) after the addition of the P300 inhibitor C646. **F** Co-IP: P300 antibody captured PKM2 and P65 in the whole-cell lysates of MSCs and CAFs (AGS exosome treated).

We showed that NF-κB P65 was bound to the promoter of the *PKM2* gene (Fig. 5A, B), indicating that NF-κB P65 upregulates the transcription of the *PKM2* gene. We also presented that PKM2 can directly bind to NF-κB P65 in CAFs compared to the MSC controls, using the Co-IP assay (Fig. 5C, D). To investigate whether these interactions happened in the nucleus, we purified the nuclear proteins and carried out the Co-IP assay. We found that PKM2 bound to NF-κB P65 in the nucleus (Supplementary Fig. 6). The nuclear translocation of PKM2 may promote the NF-κB P65 retention in the nucleus. To further confirm our estimations, we added a PKM2 nuclear translocation inhibitor (olaparib) to the CAF induction and differentiation system and then performed the western blotting analysis to detect the expression of PKM2 and P65 in the nucleus. We found that the expression of both proteins was downregulated (Fig. 6A), indicating that PKM2 nuclear translocation might promote P65 retention in the nucleus. IL-6 and IL-8 secreted by CAFs were also downregulated after the addition of olaparib (Fig. 6B).

NF-κB P65 phosphorylation was required for P65 recruitment into the nucleus. Therefore, phosphorylated P65 could only remain in the nucleus for a short time, resulting in the transient activation of transcription of the NF-κB signaling pathway and leading to the emergence of phosphorylated P65 from the nucleus. We previously confirmed that gastric cancer exosomes could upregulate NF-κB P65 phosphorylation; however, P65 acetylation was required for NF-κB P65 nuclear retention. Therefore, western blotting analysis was performed to detect the expression of acetylated P65 in the nucleus during the differentiation of MSCs into CAFs. The expression of acetylated P65 at the K310 site gradually increased with the induction time (Fig. 6C, D).

According to previous reports, NF-κB P65 might be acetylated through histone acetyltransferase P300; therefore, we detected P300 expression in the nucleus during the differentiation of MSCs into CAFs by using western blotting assay and found that P300 level also gradually increased with time (Fig. 6C, D). P300 in the nucleus was downregulated when PKM2 nuclear translocation was inhibited (Fig. 6A). The expression of acetylated P65 in the nucleus of CAF cells was downregulated after the addition of the P300 inhibitor (C646) to the CAF culture system (Fig. 6E). The results of Co-IP verified that P300 could directly bind to P65 or PKM2 (Fig. 6F). Therefore, we speculate that PKM2 promotes NF-κB P65 acetylation through P300. After inhibiting PKM2 nuclear translocation, the expression of IL-6 and IL-8 in CAFs was downregulated (Fig. 6B). Therefore, PKM2 nuclear translocation leads to acetylation of NF-κB P65, which causes P65 nucleus retention and constant activation and transcription of the NF-κB signaling pathway.

### Histone acetyltransferase P300/CBP inhibitor suppressed tumor proliferation in vivo

The previous experiment verified that PKM2 promotes NF-κB P65 acetylation through histone acetyltransferase P300. To investigate the effect of blocking the interaction between PKM2 and P65 on tumor proliferation, the histone acetyltransferase P300 inhibitor C646 was injected around the tumor in the AGS subcutaneous xenograft tumor model. The tumor volume in the AGS mixed with the MSC group was significantly higher than that in the AGS group. The tumor volume in the AGS mixed with the MSC group was significantly lower than that in the control group after C646 administration (Supplementary Fig. 7).

## Discussion

PKM2 is not only a pivotal enzyme for glycolysis but also plays a role in various nonmetabolic functions^13^. PKM2 can undergo nuclear translocation under stimulation by IL-3 and epidermal growth factor receptor^24,25^. PKM2 performs several functions in the nucleus via different circumstances. PKM2 regulates transcription via binding to transcription factors. For example, PKM2 regulates the transcription of HIF-1 target genes via binding to HIF-1α subunit in the nucleus^26^. PKM2 is also reported to promote the transcription of octamer-binding transcription factor 4 (Oct 4) target genes via binding with Oct4 in the nucleus of stem cells^27^. Furthermore, PKM2 can function as a protein kinase. It is reported that PKM2 phosphorylated signal transducer and activator of transcription 3 to activate the transcription of MEK5^28^. Finally, PKM2 can serve as a transcription factor via binding to the CCND1 promoter region^25^.

Our study showed that gastric cancer exosomes could induce the differentiation of MSCs into CAFs. NF-κB P65 was retained in the nucleus of CAFs, and the expression of their target genes such as IL-6, IL-8, MCP1, and IL-1β was upregulated. Proteins of the canonical NF-κB signaling pathway in CAFs, such as IKKβ and IKβα, showed no significant changes in total protein and phosphorylated protein levels. In the canonical NF-κB signaling pathway, phosphorylated NF-κB P65 is usually transiently translocated into the nucleus, dephosphorylated rapidly, and then degraded. Therefore, the sustained activation of NF-κB in CAFs through NF-κB P65 nuclear retention may be mediated by noncanonical pathways. PKM2 can promote the activation of the NF-κB signaling pathway in tumor cells^14,29,30^, and PKM2 overexpression can contribute to NF-κB P65 phosphorylation and thus promote autophagy. However, the mechanism by which PKM2 promotes NF-κB activation is ambiguous^31^. After treatment with gastric cancer exosomes, P65 was retained in the nucleus of CAFs, and the expression of inflammatory factors was induced. The PKM2 protein level significantly increased in the nucleus of CAFs. Therefore, we speculate that nuclear PKM2 might be involved in mediating NF-κB P65 nuclear retention of CAFs.

To verify this hypothesis, we first examined whether the upregulation of nuclear PKM2 and nuclear NF-κB P65 in CAFs was consistent. This was detected by western blotting assay and confocal microscopy. We found that the expression of these proteins was simultaneously upregulated with the progression of the effect of gastric cancer exosomes. Phosphorylation of NF-κB P65 was previously considered necessary for P65 nuclear translocation. In our previous studies, we found that AGS exosomes can induce rapid and transient NF-κB P65 phosphorylation in MSCs^18^. However, to further understand the mechanism responsible for constant P65 nuclear retention in CAFs, we examined the acetylated P65 protein, as phosphorylation and acetylation of NF-κB P65 were reported to sustain P65 nuclear retention^32^. The expression of acetylated P65 in the nucleus of CAFs differentiated from MSCs induced by gastric cancer exosomes was gradually upregulated as the induction time progressed. P65 acetylation usually relies on histone acetyltransferase P300^33–35^; therefore, we detected the expression of P300 in the nucleus of CAFs and found that P300 expression was also upregulated.

To further clarify the source of upregulated PKM2 in CAFs induced by gastric cancer exosomes, we first constructed a PKM2-eGFP fusion protein gene, transfected it into AGS cells, screened for the AGS cell line stably expressing PKM2-eGFP, extracted exosomes, and then added these exosomes to the culture system of CAFs differentiated from MSCs by gastric cancer exosomes. The PKM2-eGFP fusion protein could enter the cytoplasm and nucleus of CAFs. Therefore, the increased PKM2 protein in CAFs may be derived directly from gastric cancer exosomes. However, in previous experiments, we found that PKM2 was also upregulated at the mRNA level in CAFs; therefore, a part of the increased PKM2 protein in CAFs was also endogenous. NF-κB P65 has a binding site in the PKM2 promoter region. Xu et al. found that NF-κB P65 could directly interact with the PKM2 promoter region to promote PKM2 transcription in breast cancer^36^. Yang et al. reported that lipopolysaccharide (LPS) could promote PKM2 expression by activating the NF-κB signaling pathway in colorectal cancer and reverses TNF-α and IL-1β production and cell proliferation induced by LPS in colon cancer cells through knockout of PKM2^37^. We confirmed that NF-κB P65 directly interacts with the PKM2 promoter region through a ChIP experiment. PKM2 and P65 constitute a positive feedback loop in CAFs to promote their expression. Therefore, in the early stage, upregulated PKM2 in CAFs might be directly derived from exosomes, and after the activation of the NK-κB signaling pathway in the late stage, this positive feedback could contribute to the continuous increase in PKM2, which ultimately induces the persistent activation of the NK-κB signaling pathway.

To further understand the interaction between PKM2, P300, and P65, the Co-IP experiment was first conducted to demonstrate their direct interaction. We then added the PKM2 nuclear translocation inhibitor olaparib or the P300 inhibitor C646 to CAFs differentiated from MSCs induced by gastric cancer exosomes. We found that the expression of acetylated NF-κB P65 was downregulated under the effect of the two inhibitors, thus proving that the constant activation of the NF-κB signaling pathway in CAFs induced by gastric cancer exosomes was associated with the interaction between PKM2, P300, and P65 in the nucleus. P300 acts as a bridge between PKM2 and P65. Therefore, the P300 inhibitor C646 was injected around the tumor in the AGS subcutaneous xenograft tumor model to detect the effect of P300 inhibition on tumor proliferation. C646 significantly reduced the tumor growth in the AGS mixed with the MSC group. It was difficult to differentiate whether the blocking effect was in CAFs or tumor cells because C646 was not selective for tumor cells or CAFs. It was reported that the P300 inhibitor C646 suppressed tumor growth and promoted apoptosis in gastric cancer cells^38^. Therefore, regardless of whether the blocking effect was in CAFs or tumor cells, blocking P300 might be an effective therapeutic strategy for gastric cancer.

## Conclusion

In summary, the continuous activation of the NF-κB signaling pathway depends on PKM2 nuclear translocation in CAFs derived from gastric cancer exosome-induced MSCs. The abnormal energy metabolism of CAFs induced by tumor cells may contribute to the continuous activation of inflammatory signaling pathways, leading to the secretion of many inflammatory factors and promoting the formation of the tumor inflammatory microenvironment. In this context, our findings highlight the therapeutic potential of targeting PKM2, as it is involved in both abnormal energy metabolism and inflammatory activation in CAFs induced by tumor cells.

## Supplementary information

Supplementary Figure Legend

Supplementary Figure 1

Supplementary Figure 2

Supplementary Figure 3

Supplementary Figure 4

Supplementary Figure 5

Supplementary Figure 6

Supplementary Figure 7

## Data Availability

All the data generated or analyzed during this study are included in this published article and its supplementary information files.
