# Supplementary material for: The role of PKM2 nuclear translocation in the constant activation of the NF-κB signaling pathway in cancer-associated fibroblasts
Source: Cell Death Dis. 2021 Mar 17;12(4):291. doi: 10.1038/s41419-021-03579-x (PMC7969736; doi:10.1038/s41419-021-03579-x)
Supplement: Supplementary file 1 — Supplementary Figure Legend [file 41419_2021_3579_MOESM1_ESM.docx]

**Supplementary Fig 1. Flow cytometry detection of MSC phenotype.**

**Supplementary Fig 2. Identification of gastric cancer exosomes.** A: The morphology of GC803 and AGS exosomes detected by transmission electron microscopy. B: The specific biomarker expression of GC803 and AGS exosomes detected by western blotting assay.

**Supplementary Fig 3. Comparison of adipogenic and osteogenic differentiation ability between MSCs and CAFs.**

**Supplementary Fig 4. GC803 exosomes induced differentiation of MSCs into CAFs.** A: Western blotting analysis detected that CAF-specific protein expression was upregulated after MSCs were induced by GC803 exosomes. B: Quantitative PCR analysis detected that the expression of inflammatory factors and chemokines was upregulated in CAFs differentiated from MSCs after treatment with GC803 exosomes. C: Western blotting analysis detected the protein expression of the NF-κB signaling pathway in CAFs differentiated from MSCs after treatment with GC803 exosomes.

**Supplementary Fig 5. Confocal microscopy detected PKM2 and P65 expression in the nucleus during the differentiation of MSCs into CAFs induced by GC803 exosomes.** Blue: Nucleus (Hoechst 33342). Red: P65 (rhodamine). Green: PKM2 (Alexa Fluor 488).

**Supplementary Fig 6: A.** Western blotting analysis detected the nuclear lysate protein captured by Co-IP P65 antibody containing PKM2 protein. B: Western blotting analysis detected nuclear lysate protein captured by Co-IP PKM2 antibody containing P300 protein.

**Supplementary Fig 7. The effect of the histone acetyltransferase P300 inhibitor (C646) on the tumor volume of the AGS subcutaneous xenograft tumor model.** The tumor volumes of three group of mice were compared at different time point (n=5, left). The tumor sizes of three groups at week 7 after injections were presented (n=5, right).
