# Supplementary figures and images for: The role of PKM2 nuclear translocation in the constant activation of the NF-κB signaling pathway in cancer-associated fibroblasts
Source: Cell Death Dis. 2021 Mar 17;12(4):291. doi: 10.1038/s41419-021-03579-x (PMC7969736; doi:10.1038/s41419-021-03579-x)

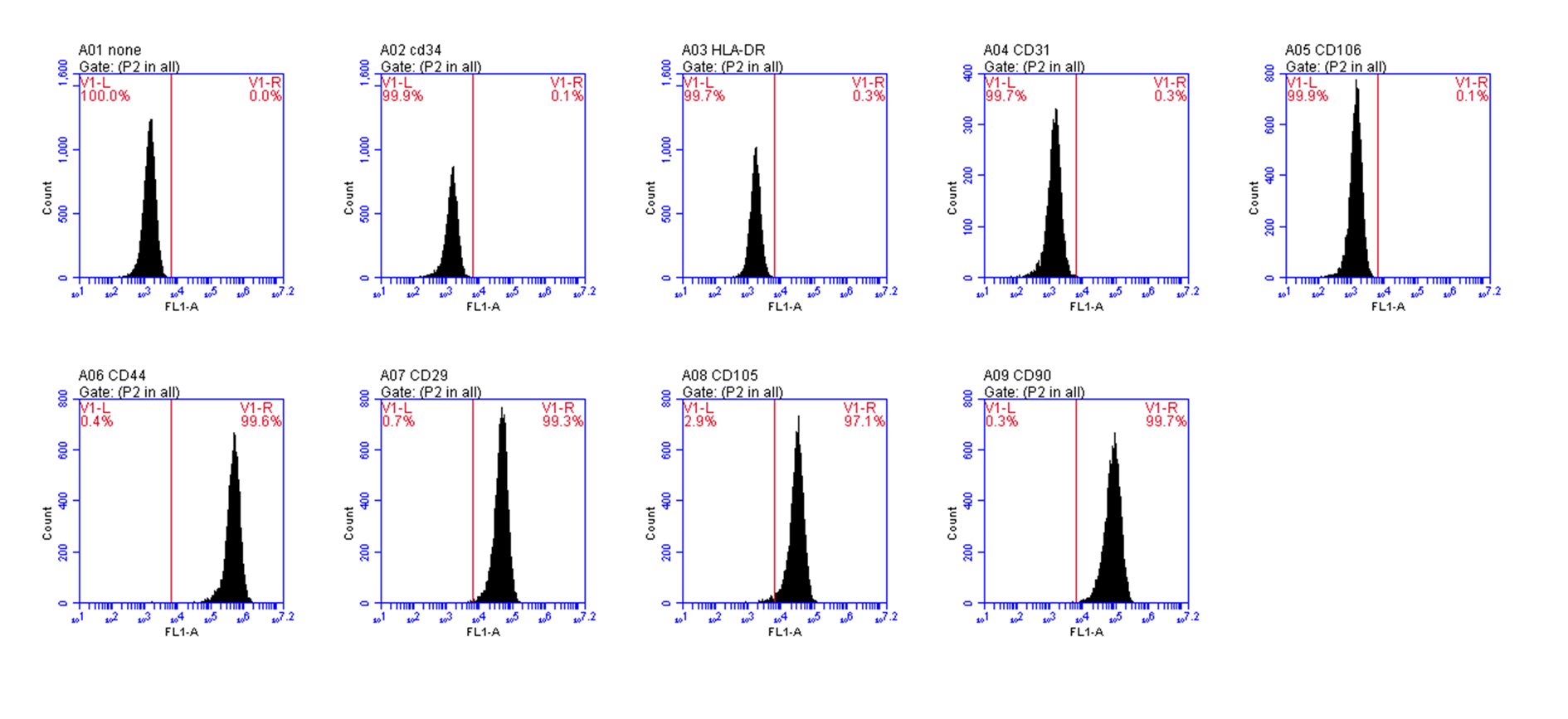

Supplement: Supplementary file 2 — Supplementary Figure 1 [file 41419_2021_3579_MOESM2_ESM.tif]

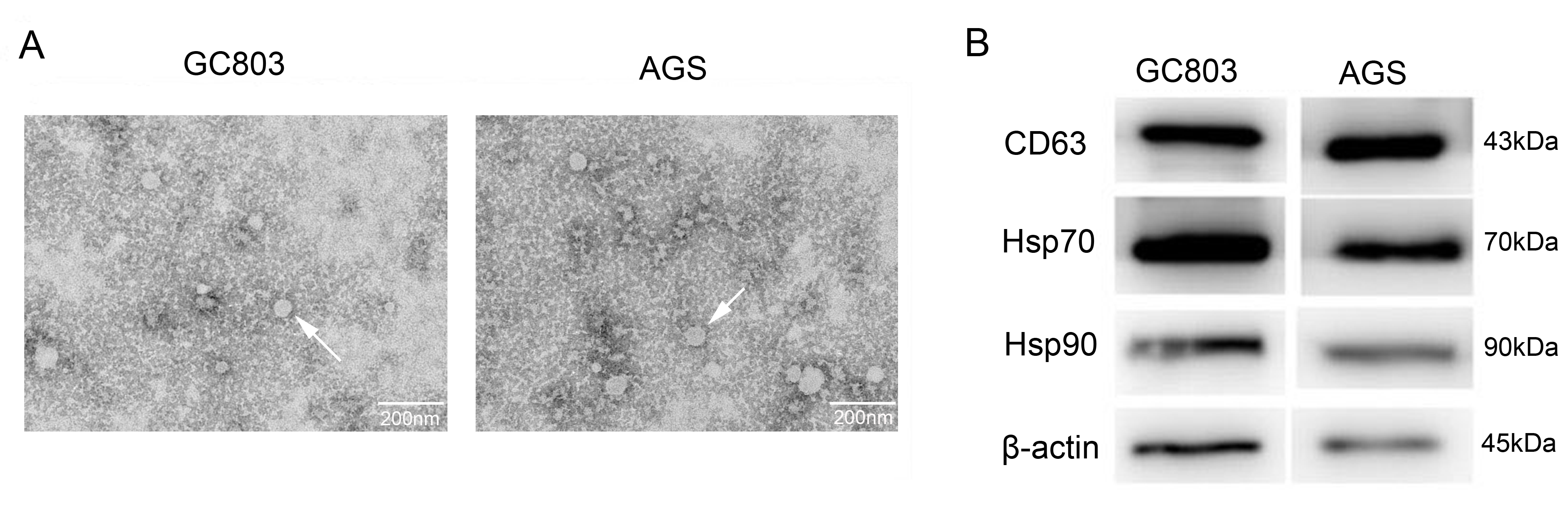

Supplement: Supplementary file 3 — Supplementary Figure 2 [file 41419_2021_3579_MOESM3_ESM.tif]

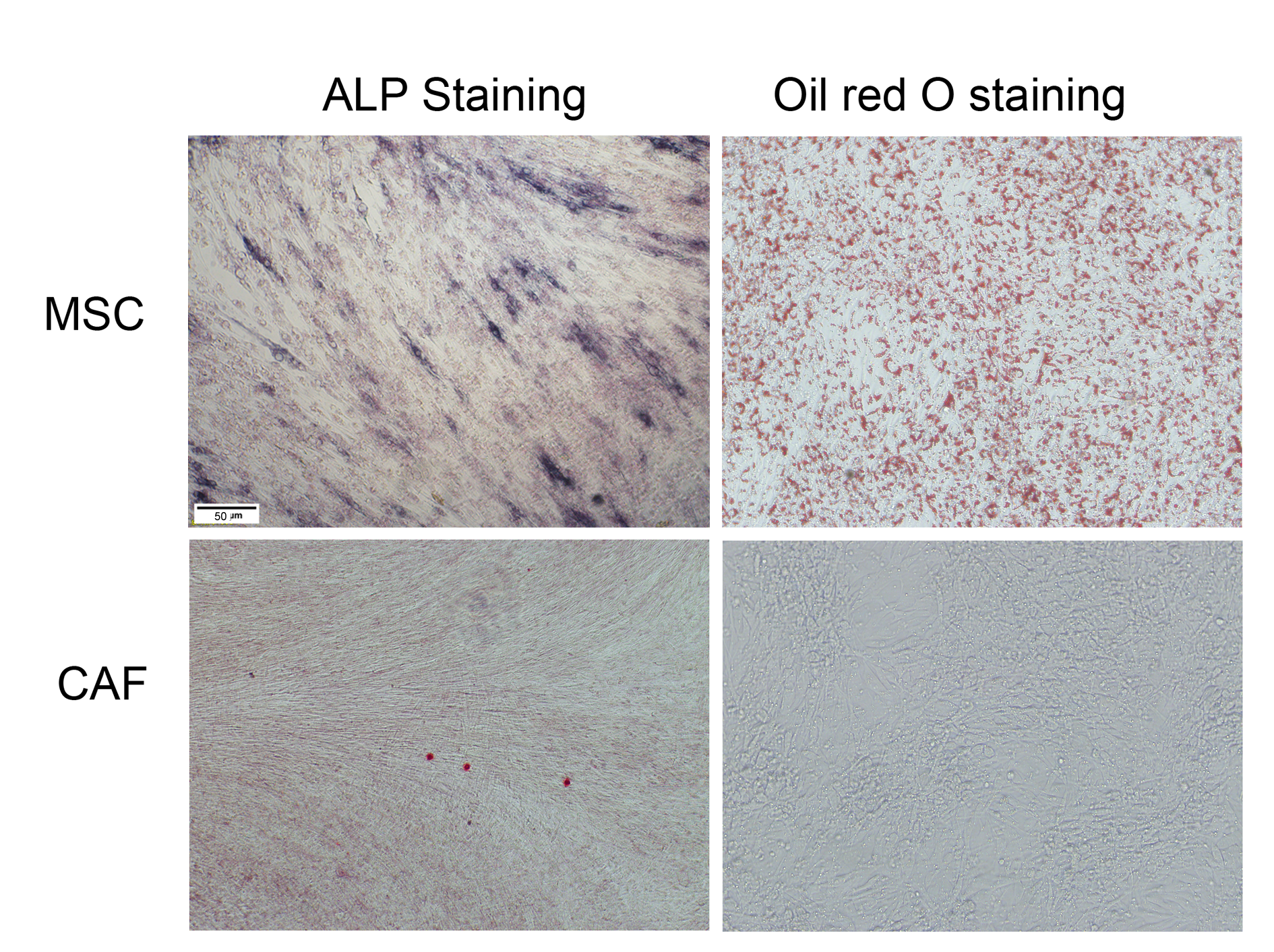

Supplement: Supplementary file 4 — Supplementary Figure 3 [file 41419_2021_3579_MOESM4_ESM.tif]

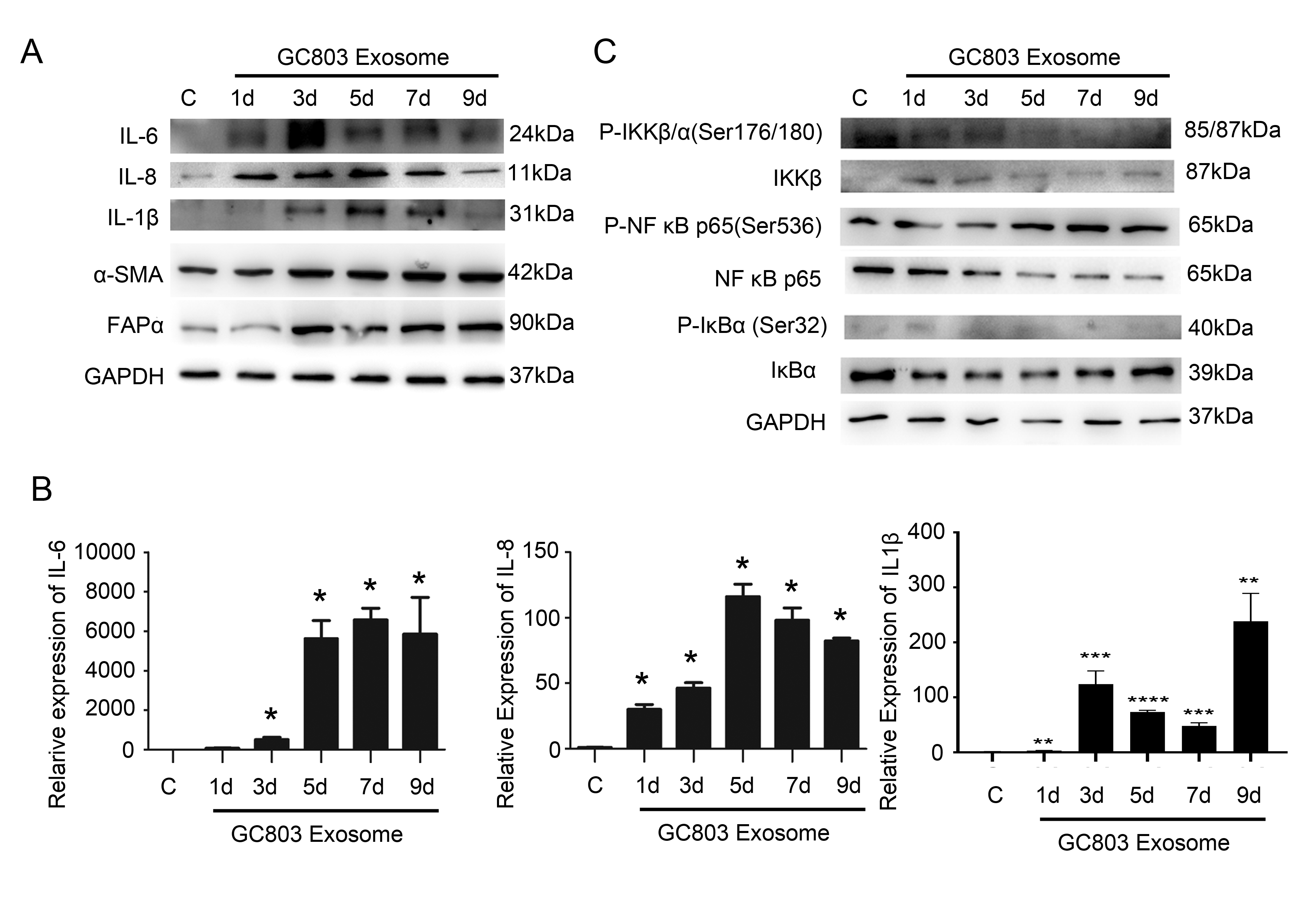

Supplement: Supplementary file 5 — Supplementary Figure 4 [file 41419_2021_3579_MOESM5_ESM.tif]

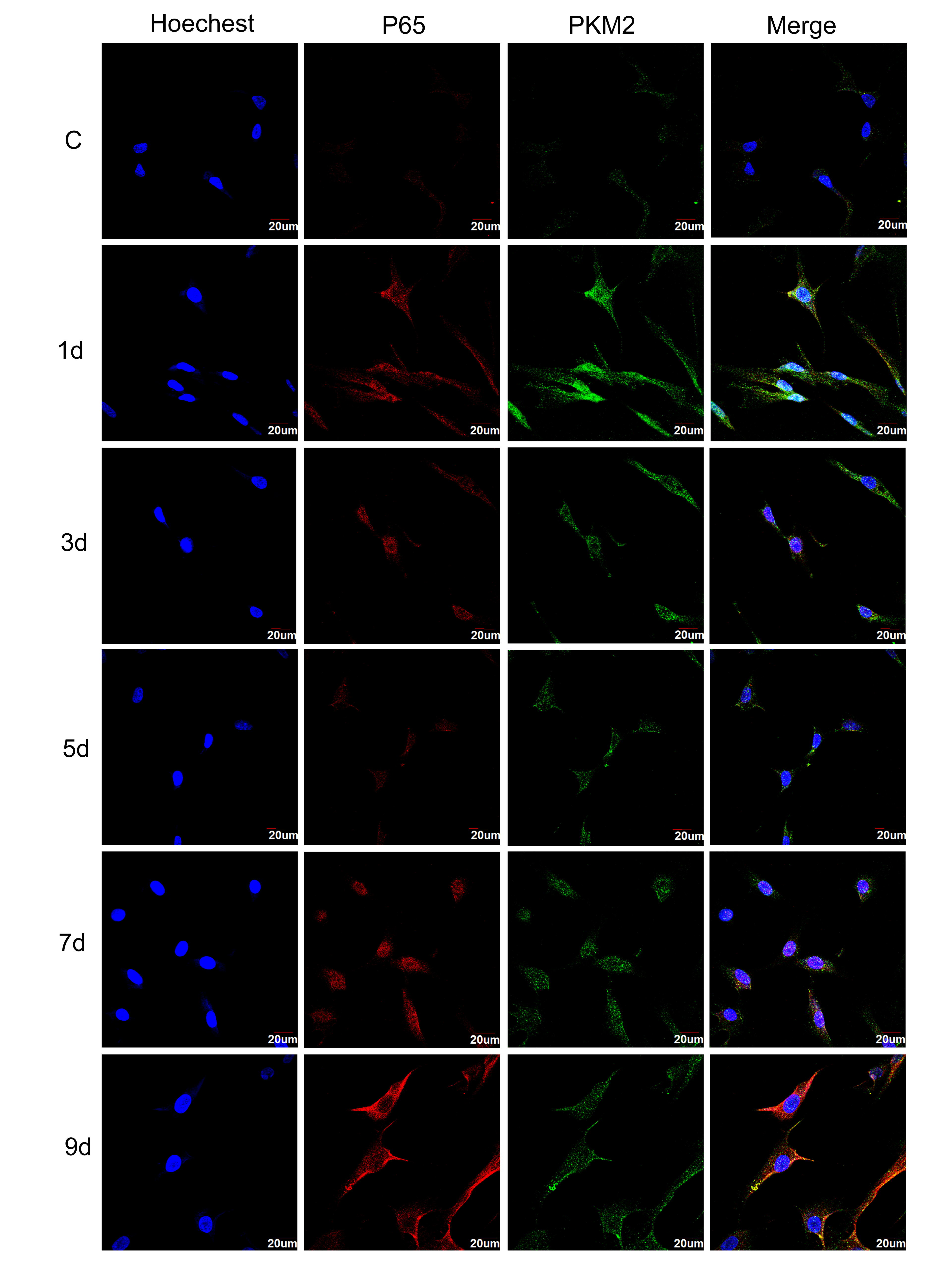

Supplement: Supplementary file 6 — Supplementary Figure 5 [file 41419_2021_3579_MOESM6_ESM.tif]

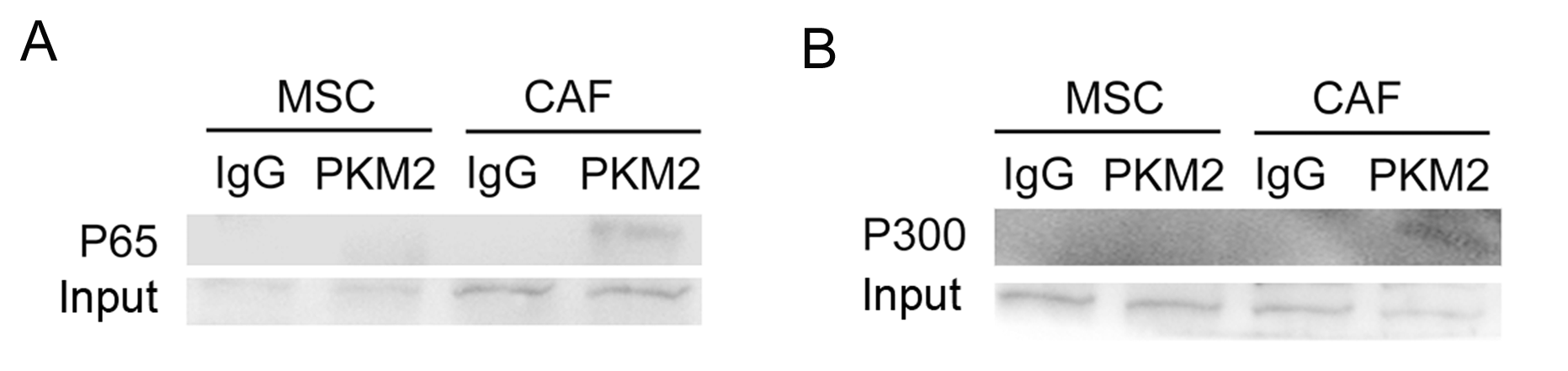

Supplement: Supplementary file 7 — Supplementary Figure 6 [file 41419_2021_3579_MOESM7_ESM.tif]

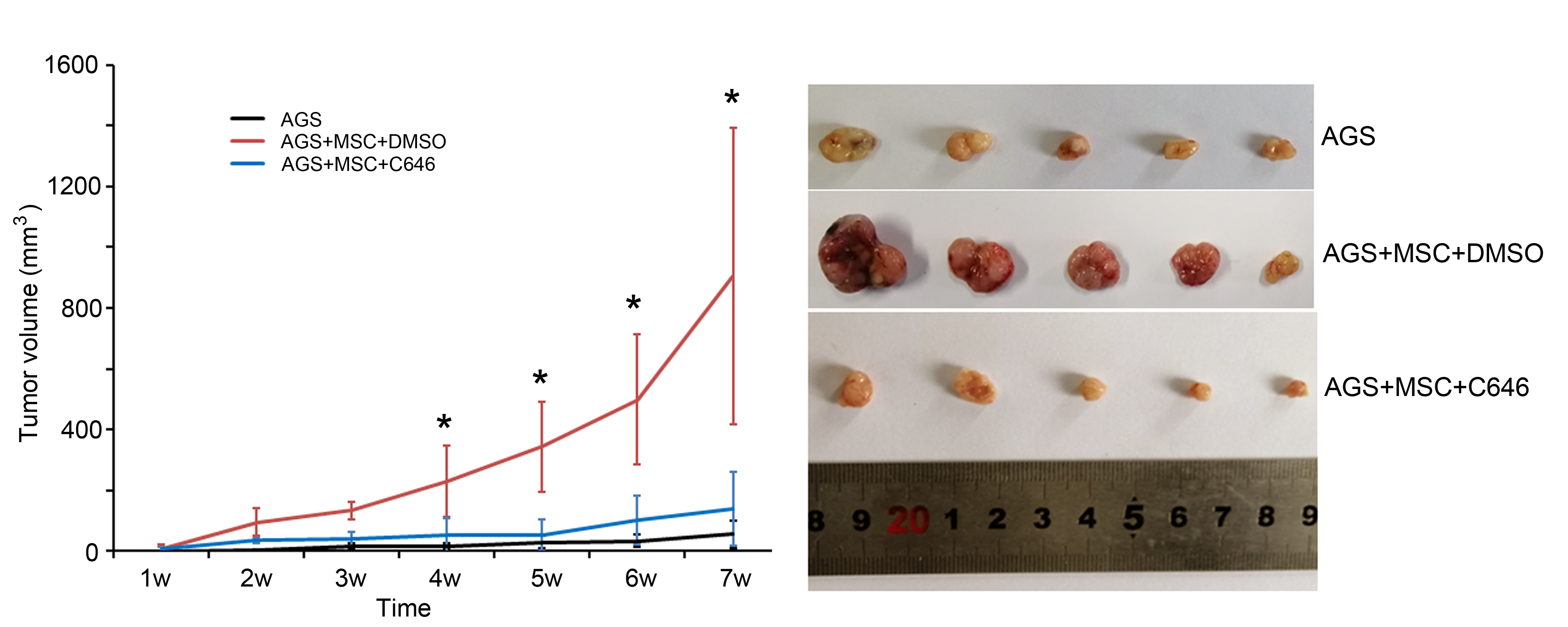

Supplement: Supplementary file 8 — Supplementary Figure 7 [file 41419_2021_3579_MOESM8_ESM.tif]
